# Case Report: Retrospective surgeon-guided reassessment of clinical target volume delineation in low-dose radiotherapy for postoperative chylothorax: an eight-patient case series

**DOI:** 10.3389/fmed.2026.1828683

**Published:** 2026-06-02

**Authors:** Mu-Chou Lin, Forn-Chia Lin, Ying-Yuan Chen

**Affiliations:** 1 Division of Thoracic Surgery, Department of Surgery, National Cheng Kung University Hospital, College of Medicine, National Cheng Kung University, Tainan, Taiwan; 2 Department of Radiation Oncology, National Cheng Kung University Hospital, College of Medicine, National Cheng Kung University, Tainan, Taiwan

**Keywords:** chylothorax, clinical target volume, postoperative, radiotherapy, thoracic duct ligation, treatment outcome

## Abstract

**Background:**

Postoperative chylothorax is an uncommon but challenging complication after thoracic surgery. Low-dose external beam radiotherapy (RT) has been proposed as an adjunct treatment when conservative management or surgical intervention fails, but factors associated with treatment success remain unclear.

**Methods:**

We conducted a retrospective case series of eight patients with persistent postoperative chylothorax treated with RT at a single institution (January 2019–July 2022). All patients received low-dose RT (9 Gy in 1.5-Gy fractions or 10 Gy in 2-Gy fractions). To explore factors associated with outcomes, thoracic surgeons retrospectively reviewed surgical dissection fields and re-contoured clinical target volumes (CTVs), which were compared with delivered RT isodose distributions.

**Results:**

Chylothorax resolved without recurrence in 4 of 8 patients (50%). Patients with extremely high baseline chyle output (e.g., >1,500 mL/day) showed limited response to RT despite apparently adequate overall isodose coverage. Retrospective plan review also demonstrated incomplete inclusion of surgically dissected lymphatic regions within the RT field in several unsuccessful cases.

**Conclusion:**

This case series highlights the value of retrospective surgeon-guided CTV reassessment in identifying clinically relevant coverage gaps in postoperative chylothorax treated with low-dose RT. In this series, patients with high output (>1,500 mL/d) had poor responses to RT, suggesting that earlier surgical intervention may be warranted in selected cases; however, this observation requires validation in larger cohort.

## Introduction

Chylothorax is the accumulation of chyle within the pleural space and may occur spontaneously or after trauma and surgery. Postoperative chylothorax is most commonly associated with esophageal and mediastinal surgery, with a reported incidence of up to 4% following esophagectomy (1). Although uncommon, it can lead to prolonged hospitalization and substantial morbidity, including respiratory complications, malnutrition, sepsis, and mortality.

Initial management involves dietary restriction and parenteral nutrition, with surgical thoracic duct ligation considered when conservative treatment fails (2). Lymphangiography with or without embolization is another option but may be limited by technical availability (3). Low-dose external beam radiotherapy (RT) has been reported as a noninvasive adjunct for refractory cases (4–7); however, target definition is challenging because a discrete leakage site is often not visualized. Because there is no visible target on imaging, we hypothesize that RT failures are frequently due to inadequate target delineation, which may be mitigated by surgeon-guided mapping of the operative bed. Here, we present a retrospective root-cause analysis of an eight-patient case series treated with low-dose RT, highlighting the use of surgeon-guided CTV reassessment based on operative dissection fields to identify clinically relevant coverage gaps observed in unsuccessful cases.

## Case description

### Patients and diagnostic assessment

We present an eight-patient case series of postoperative chylothorax treated with low-dose radiotherapy (RT) at our hospital between January 2019 and July 2022. Patient characteristics, surgical indications, and perioperative courses are summarized in Table 1. The median age was 55 years (range, 39–67 years). Surgical indications included esophageal squamous cell carcinoma (n = 3), lung adenocarcinoma (*n* = 2), invasive thymoma (*n* = 2), and benign mediastinal lymphadenopathy (n = 1). Seven patients underwent minimally invasive thoracic surgery and one underwent median sternotomy.

**Table 1:** Patient characteristics.

Patient	Diagnosis	Surgery	MLND	Prior intervention	Chylothorax to RT (day)	TG level (mg/dL)	Chest drainage before RT (ml/day)	Delivered RT dose (Gy)	Chest drainage at the end of RT(ml/day)	Day of chest tube removal	TG level (mg/dL) before chest tube removal	Further management
Patient 1	Para-aortic LAP	VATS LN excision	N/A	NPO, Bilateral TDL(x3), TDE	71	713	184	9	112	7	Not available	
Patient 2	Thymoma with MG	Sternotomy thymothymectomy	N/A	NPO only	9	1,404	230	5	645	22	294	Right TDL
Patient 3	LADC	VATS RS3 segmentectomy	Station 2, 4, 13	Low fat diet only	25	2,299	180	10.8	20	16	39	
Patient 4	ESCC	VATS three-field (McKeown) esophagectomy	Station 7, 8 U, right and left RLN	NPO only	7	1760	655	10.8	15	10	Not available	
Patient 5	Lymphoepithelioma-like carcinoma	VATS RLL	Station 2, 4, 7, 9, 10, 11	NPO, bilateral TDL	12	Not available	2,810	9	1,380	28	52	Bilateral TDL + pleurodesis
Patient 6	Esophageal adenocarcinoma	VATS three-field (McKeown) esophagectomy	Station 7, 8 U, right and left RLN	NPO, Right TDL	13	264	360	50	20	15	46	
Patient 7	ESCC	VATS three-field (McKeown) esophagectomy	Station 7, right and left RLN	NPO only	9	Not available	1730	10	2,450	13	21	Left TDL
Patient 8	Thymoma with nephrotic syndrome	VATS left thymothymectomy	Station 10	NPO only	10	334	550	10.8	320	28	Not available	Left TDL

ESCC, esophageal squamous cell carcinoma; LAP, lymphadenopathy; LN, lymph node; NPO, nil per os; MG, myasthenia gravis; RLN, recurrent laryngeal nerve; TDE, thoracic duct embolism; TDL, thoracic duct ligation; TG, triglyceride.

Chylothorax was diagnosed based on the characteristic milky appearance of pleural drainage and, when necessary, confirmed by elevated triglyceride levels (>110 mg/dL) in pleural effusion. Lymphangiography was performed in six patients, but a definitive leakage site could not be localized in all cases.

In our clinical practice, the diagnosis of chylothorax was primarily established by the characteristic milky appearance of the pleural effusion after a normal diet. Treatment success was defined by the transition to clear serous drainage under a regular diet. Consequently, biochemical indicators such as triglyceride levels or lymphocyte counts were not routinely performed for all patients if the clinical presentation and resolution were definitive.

The primary indication for RT was persistent chylothorax in which conservative management was insufficient or when patients were reluctant to undergo early re-operation due to the physical stress of the recent index surgery. RT provided a non-invasive alternative to potentially avoid the morbidity of repeated thoracic surgery. During this three-year period, a standardized treatment protocol was not yet established. Instead, treatment was guided by Shared Decision-Making (SDM). Patients were informed of the options, including conservative management with adjunctive RT or direct surgical intervention (TDL). RT was also utilized as a salvage therapy for those who had failed prior surgical or interventional procedures. For patients with manageable drainage volumes, RT offered the advantage of being performable in an outpatient setting.

### Therapeutic interventions

All patients received low-dose external beam RT delivered as either 9 Gy in 1.5-Gy fractions or 10 Gy in 2-Gy fractions, administered once daily over six or five consecutive days. Two patients received RT concurrently with postoperative adjuvant therapy for thymoma or esophageal cancer (50 Gy in 25 fractions).

A no-fat enteral or parenteral diet was maintained until RT completion. If pleural drainage decreased to <150 mL/day with a clear serous appearance, a regular diet was resumed on post-RT day 1, followed by chest tube removal if drainage remained stable. Before RT, Patient 1 underwent bilateral thoracic duct ligation (TDL) combined with thoracic duct embolization, and Patients 5 and 6 underwent ipsilateral VATS-guided TDL; however, these interventions failed to control chylothorax. The remaining patients received conservative management only (nil per os or low-fat diet) before RT.

### Timeline of the episode of care

**Figure 1:**
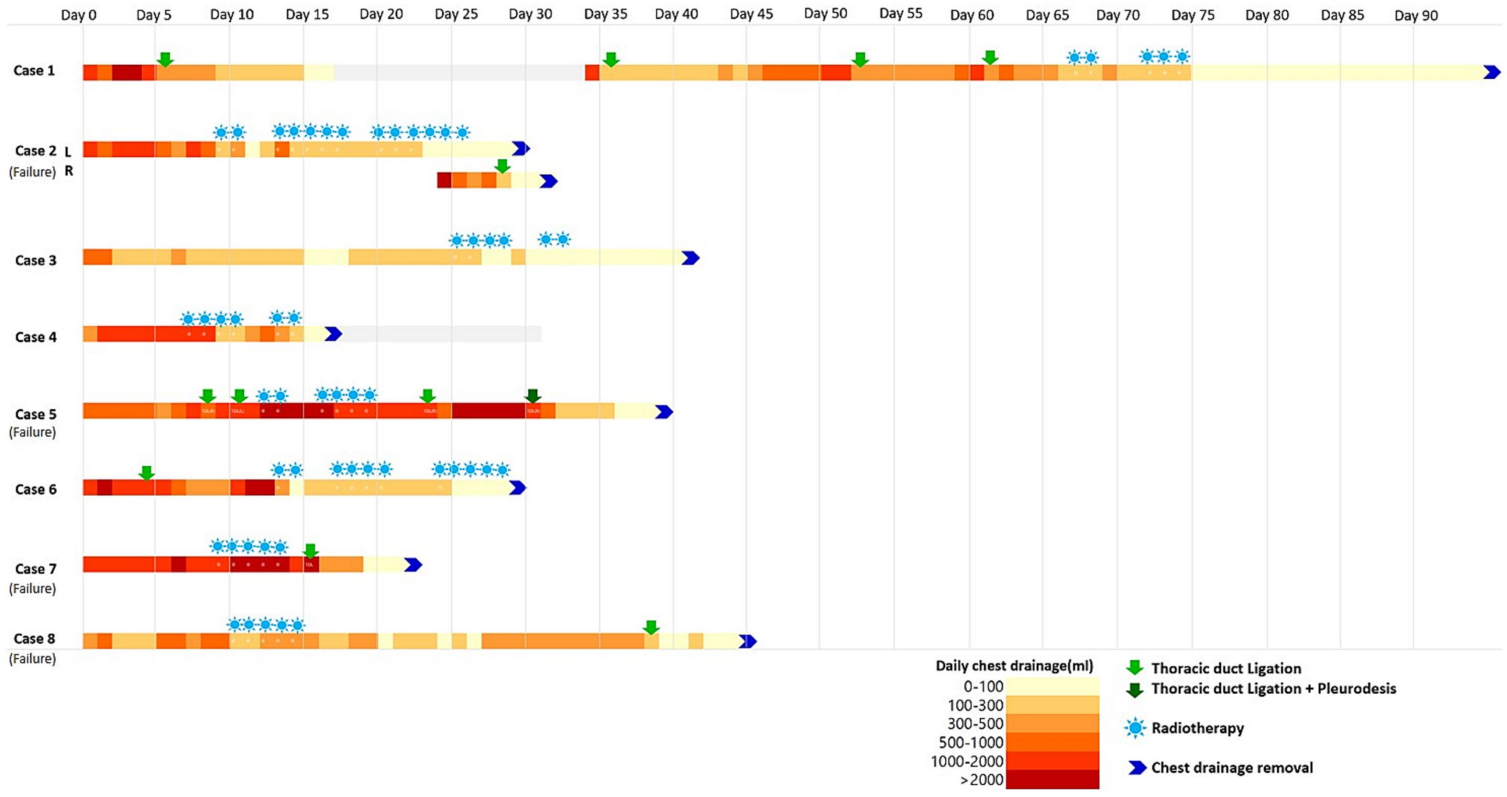
Time-aligned heat map of daily chest tube drainage volumes for eight postoperative chylothorax cases from postoperative day 0 to 90. Colors indicate drainage output strata, and symbols denote thoracic duct ligation (± pleurodesis), radiotherapy courses, and chest tube removal. Left and right sides are displayed separately for case 2.

The overall treatment course and daily drainage trajectories are shown in Figure 1. RT was administered concurrently with initial dietary control in five patients and as salvage therapy after failed interventions in three patients. The median interval between chylothorax diagnosis and RT initiation was 11 days (range, 7–71 days). At RT initiation, daily drainage volumes ranged from 160 to 880 mL/day and decreased to 20–244 mL/day by RT completion.

### Outcomes and follow-up

RT resolved chylothorax in four patients (Patients 1, 3, 4, and 6), yielding an overall success rate of 50%. Four patients (Patients 2, 5, 7, and 8) required additional intervention, including TDL.

Regarding safety, no acute or late radiotherapy-related complications, such as radiation pneumonitis, esophagitis, or skin dermatitis, were observed in any of the eight patients. This favorable safety profile may be related to the low RT doses (9–10 Gy) utilized in this series.

For the four patients who failed to respond to RT (Patients 2, 5, 7, and 8), salvage thoracic duct ligation (TDL) was performed. The surgical technique involved a “mass ligation” approach, where all soft tissue between the aorta and the spine was ligated to ensure complete interruption of lymphatic flow. During these salvage operations, we specifically inspected high-risk areas from previous dissections, such as the paratracheal and subcarinal regions; however, no visible or discrete leakage sites were identified. Notably, Patient 2’s initial left-sided chylothorax resolved after RT, but a subsequent right-sided leak necessitated right-sided TDL. Patient 5 experienced a more complex course, requiring sequential bilateral TDL followed by right-sided pleurodesis to eventually achieve resolution. No patient experienced recurrence of chylothorax during the six-month follow-up period after discharge.

**Figure 2:**
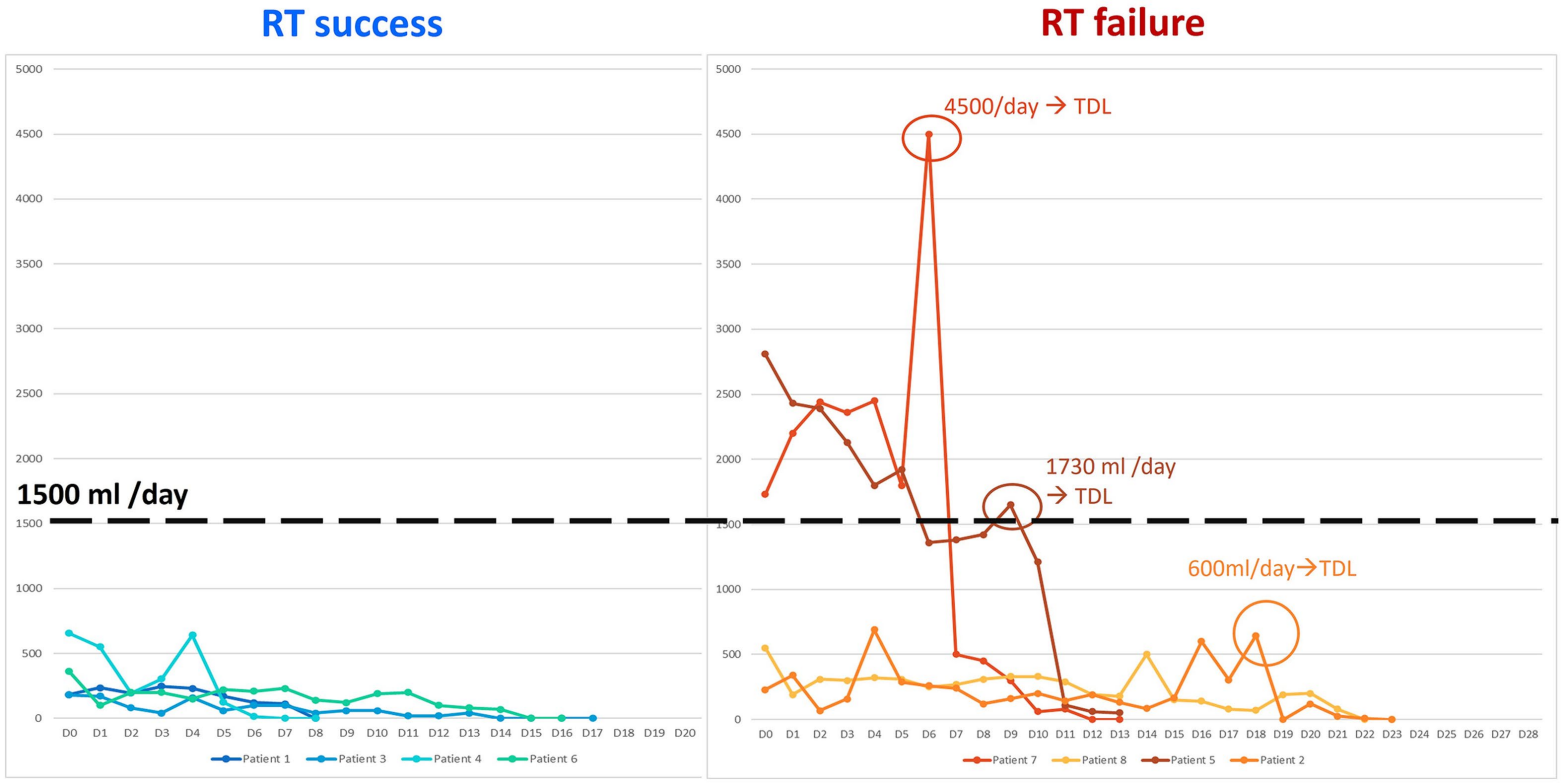
Daily chest-tube drainage trajectories during radiotherapy (RT) in patients with successful (left panel) and unsuccessful (right panel) outcomes. The dashed line indicates the 1,500 mL/day reference level. Symbols mark thoracic duct ligation (TDL) when performed.

Figure 2 compares chest drainage volume trends between patients with successful and unsuccessful outcomes. In the successful group, all patients demonstrated a gradual and sustained reduction in drainage volume. In contrast, two patients in the unsuccessful group (Patients 5 and 7) presented with markedly high initial drainage volumes exceeding 1,500 mL/day (2,810 and 1730 mL/day, respectively).

### Surgeon-guided CTV review and anatomical coverage assessment

Because a discrete leakage site is often not identified in postoperative chylothorax, we recognized a need for a more standardized anatomical target definition based on surgical dissection fields. During the initial treatment phase of this series, however, delineation strategies remained heterogeneous due to the lack of a predefined institutional protocol. For instance, Patients 1, 4, and 7 were planned through direct surgeon consultation, while Patients 2 and 6 followed standard adjuvant radiotherapy volumes for malignancy. For the remaining cases, volumes were determined individually by various radiation oncologists according to their clinical judgment.

To resolve these inconsistencies and identify potential reasons for treatment failure, we conducted a retrospective analysis using a standardized surgeon-guided CTV review process. In this workflow, the thoracic surgeon (YYC) utilized the original simulation CT and re-contoured the target volumes directly on the RT planning system. The surgeon individualized the CTV for each patient to meticulously encompass the specific regions of surgical dissection where lymphatic vessels were most likely injured, such as the mediastinal nodal stations or thymic horn areas. This re-delineation was performed by cross-referencing preoperative and postoperative CT scans, guided by surgical records and intraoperative landmarks—including surgical clips and sutures—as anatomical guides. A 0.7–1.0 cm margin was then applied to the CTV to account for RT uncertainties.

These surgeon-contoured CTVs were then validated by a radiation oncologist (FCL) to ensure clinical and technical feasibility, with any differences in target definition resolved by consensus. Finally, these re-contoured CTVs were overlaid with the original delivered isodose distributions to assess the anatomical completeness of coverage and identify any clinically relevant gaps—specifically, surgical dissection areas that had been excluded from the initial treatment fields.

**Figure 3:**
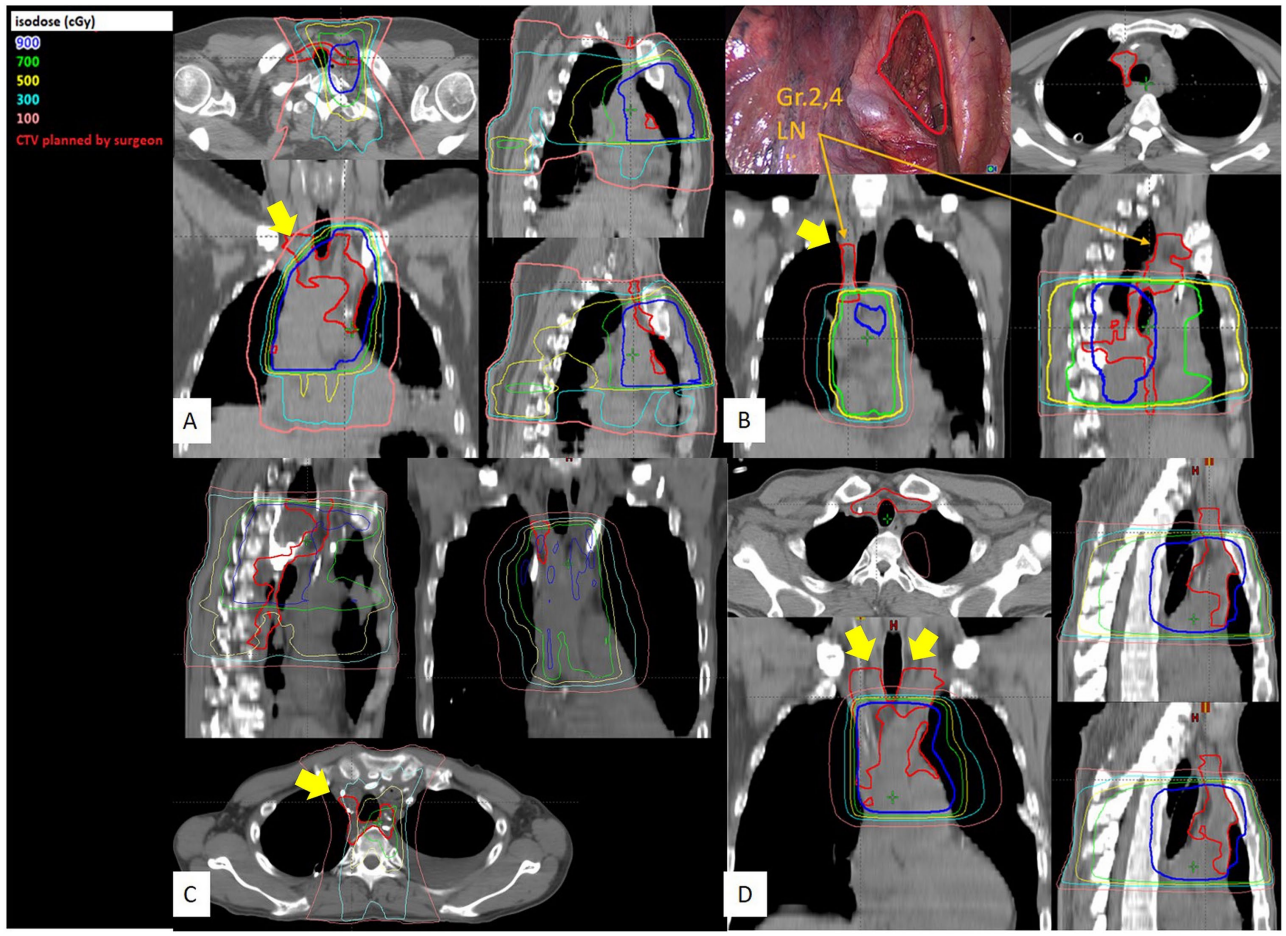
Overlay of delivered isodose maps and surgeon-planned clinical target volumes for four unsuccessful cases showing uncovered dissected regions (marked with arrows): **(A)** Patient 2: the right thymic horn (arrow) outside the 9 Gy isodose; **(B)** Patient 5: paratracheal stations 2R/4R (arrows) outside all isodoses; **(C)** Patient 7: the right recurrent laryngeal nerve corridor (arrow) outside the 9 Gy isodose; **(D)** Patient 8: bilateral thymic horns (arrows) outside all isodoses.

Supplementary Figure S1 and Supplementary Table S1 summarize coverage rates at different isodose levels; however, no consistent association was observed between overall coverage percentage and treatment outcome. In contrast, anatomical review identified clinically relevant coverage gaps in unsuccessful cases (Figure 3). In Patients 2 and 8, the bilateral thymic horns were excluded from the CTV and lay outside the 100 cGy isodose line. The radiation dose delivered to the bilateral recurrent laryngeal nerve region and the lower para-esophageal area of Patient 7 was insufficient. In Patient 5, lymph node stations 2 and 4 were omitted from the clinical target volume, resulting in complete exclusion of these regions from the RT field.

## Discussion

In this case series, low-dose RT achieved resolution of postoperative chylothorax in four of eight patients. Review of the RT treatment fields suggested that treatment success was not determined by the overall isodose coverage rate alone. Rather, adequate inclusion of surgically dissected and potentially leaky lymphatic regions within the RT treatment field appeared to be more important. These observations suggest that anatomical completeness of target coverage may play an important role in the effectiveness of RT for postoperative chylothorax.

Postoperative chylothorax remains a challenging complication. Thoracic duct ligation (TDL) is generally considered the most effective treatment, with reported success rates of 80–90% (8). However, anatomical variability may limit surgical predictability, as the thoracic duct follows a typical anatomical course in only 40–60% of patients (9–11). The evolution of our treatment strategy was rooted in the successful outcome of patient 1, our most refractory case. Following that experience, we integrated RT into our clinical options. The choice between early RT and direct salvage surgery was largely driven by patient preference and clinical stability. While our unstratified analysis shows an overall 50% success rate, the numerically higher success in the non-salvage group suggests that earlier intervention with RT might be more effective before the development of complex lymphatic collaterals or extremely high-flow states. In our cohort, chylothorax persisted in Patient 1 despite three TDL procedures and one thoracic duct embolization but was eventually resolved with low-dose external beam RT. Based on this experience, seven additional patients were treated with RT for postoperative chylothorax. The overall success rate in our series was 50%, lower than the 60–100% reported previously (7, 12–14). This difference may partly reflect variations in CTV delineation strategies and the inclusion of patients with very high chyle output in our cohort, which may represent more challenging clinical scenarios than those described in earlier reports. Together, these findings suggest that both the extent of target coverage and the magnitude of chylous leakage may influence RT response.

The first report of RT for postoperative chylothorax appeared in 2013. Sziklavari et al. (7) successfully treated seven postoperative cases using low-dose RT combined with dietary restriction, delivering 6–12 Gy to the posterior mediastinum (T3–T10) adjusted to the resected lobe. Wiesner et al. (12) reported cessation of chylous output in six of 10 patients treated with sclerosing RT, although they did not describe CTV delineation. The therapeutic effect of low-dose RT is thought to result from radiation-induced lymphatic endothelial edema and partial channel obstruction, similar to effects observed in the microvasculature (13–16).

Our lower success rate compared with Sziklavari et al. (7) and Wiesner et al. (12) likely reflects not only the small sample size of our series but also important differences in patient selection and target definition. Sziklavari et al. (7) treated postoperative chylothorax using a relatively standardized posterior mediastinal field extending from T3 to T10, adjusted to the resected lobe, and initiated RT when daily chyle flow remained >450 mL/day despite dietary restriction. In contrast, our cohort included more heterogeneous operative procedures, including esophagectomy, thymectomy, and pulmonary resection, with correspondingly different lymphatic disruption patterns and less standardized RT field design during the initial treatment period. It is therefore plausible that the favorable outcomes in Sziklavari et al. (7) were partly related to a more uniform target concept and a more homogeneous postoperative population.

Wiesner et al. (12) approached chylothorax within a broader multimodal treatment framework and recommended that treatment escalation be guided by etiology, clinical course, and drainage volume. In their series, high-output cases and many postoperative cases were preferentially directed toward earlier operative revision, while sclerosing RT achieved cessation of chylous output in 6 of 10 treated patients. However, they did not describe how RT target volumes were delineated, which limits interpretation of whether their RT outcomes were influenced by anatomical field design. This distinction is particularly relevant to our study, because our retrospective surgeon-guided review suggests that treatment failure may depend less on overall isodose coverage percentage than on whether all surgically disrupted lymphatic regions are anatomically encompassed.

Compared with TDL, RT offers the practical advantage of being non-invasive and may help avoid early reoperation in selected patients who are clinically fragile or reluctant to undergo another thoracic procedure. However, RT also has important limitations: its effect is not immediate, target definition is challenging when no discrete leak is visualized, and its efficacy may be limited in very high-output leaks. By contrast, TDL remains the standard definitive intervention, particularly in patients with high-output chylothorax or persistent postoperative leakage requiring prompt control. In this context, we consider RT not as a replacement for TDL, but as a complementary option whose success may depend on careful patient selection and anatomically appropriate target coverage.

Clinical target volume delineation is a major issue in RT for postoperative chylothorax. Unlike malignancy, postoperative chylothorax lacks a visible imaging target, and no formal guidelines exist for CTV definition. A notable aspect of our approach is the incorporation of surgeon-guided CTV delineation based on the actual surgical dissection fields. In our series, all four RT failures were associated with incomplete coverage of surgically dissected lymphatic regions, including thymic horns in thymectomy cases and omitted mediastinal nodal stations following lung resection. These findings support close collaboration between surgeons and radiation oncologists during target definition.

The clinical rationale for attempting RT in patients with high-output leaks (>1,500 mL/day) in this series was primarily guided by Shared Decision-Making (SDM). Because postoperative patients often experience significant physical and psychological stress from their index surgery, many express a strong reluctance to undergo immediate re-operation. In these scenarios, RT was utilized as a non-invasive adjunct to strict dietary restriction in an attempt to maximize the success rate of conservative management. Since our institution lacked a standardized protocol during the study period, these cases reflect an individualized clinical approach rather than a prospective trial. However, our observations suggest that massive, high-flow leaks may overwhelm the therapeutic effect of RT-induced lymphatic sclerosis. In our cohort, two patients with initial chyle outputs exceeding 1,500 mL/day failed to respond to RT despite having apparently adequate CTV coverage. While this poor response suggests that such patients may need to be prioritized for early surgical intervention—reinforcing traditional surgical dogma—this observation should be interpreted with caution due to the small sample size and requires further validation with larger cohorts. From a practical standpoint, we suggest that while strict dietary restriction remains the initial management, early TDL should be considered if output remains >1,500 mL/day, with RT reserved as an adjunctive or salvage therapy once the leakage volume has been reduced (Supplementary Figure S2).

The optimal RT dose remains uncertain. We adopted a total dose of 9 Gy based on previous reports (7, 12); higher doses did not consistently improve outcomes in our series. This case series is limited by its retrospective nature, heterogeneous RT planning strategies, variable timing of treatment initiation, and small sample size. Because of the limited number of patients, statistical analysis was not feasible, and the observed associations should therefore be interpreted with caution. Our findings should be considered hypothesis-generating clinical observations rather than definitive evidence regarding the effectiveness of radiotherapy or the impact of CTV coverage. Nonetheless, subsequent successful treatment of two additional thymectomy-related bilateral chylothorax cases using comprehensive CTV coverage further supports the potential importance of meticulous target delineation (Supplementary Figure S3).

In summary, low-dose radiotherapy may serve as a useful adjunct treatment option for postoperative chylothorax. Our observations suggest that adequate inclusion of surgically dissected lymphatic regions within the RT target volume may be important for treatment success.

## Conclusion

In summary, retrospective surgeon-guided reassessment of CTVs may help identify clinically relevant coverage gaps in postoperative chylothorax treated with low-dose RT. Our findings suggest that anatomical completeness of coverage of surgically disrupted lymphatic regions may be more important than overall isodose percentage alone. Very high-output chylothorax may have limited response to RT and may warrant earlier surgical prioritization, although this observation requires validation in larger cohorts.

## Patient perspective

Patient perspectives were not systematically collected as part of this retrospective case series because the cases were identified and reviewed retrospectively from routine clinical records. However, written informed consent for publication was obtained from all participants or their legal representatives.

## Data Availability

The original contributions presented in the study are included in the article/Supplementary material, further inquiries can be directed to the corresponding author/s.
