# Supplementary material for: Case Report: Retrospective surgeon-guided reassessment of clinical target volume delineation in low-dose radiotherapy for postoperative chylothorax: an eight-patient case series
Source: Front Med (Lausanne). 2026 Jun 2;13:1828683. doi: 10.3389/fmed.2026.1828683 (PMC13269024; doi:10.3389/fmed.2026.1828683)
Supplement: Supplementary file 1 [file Table_1.docx]

Supplementary Material

# Supplementary Figures and Tables

## Supplementary Figures

##
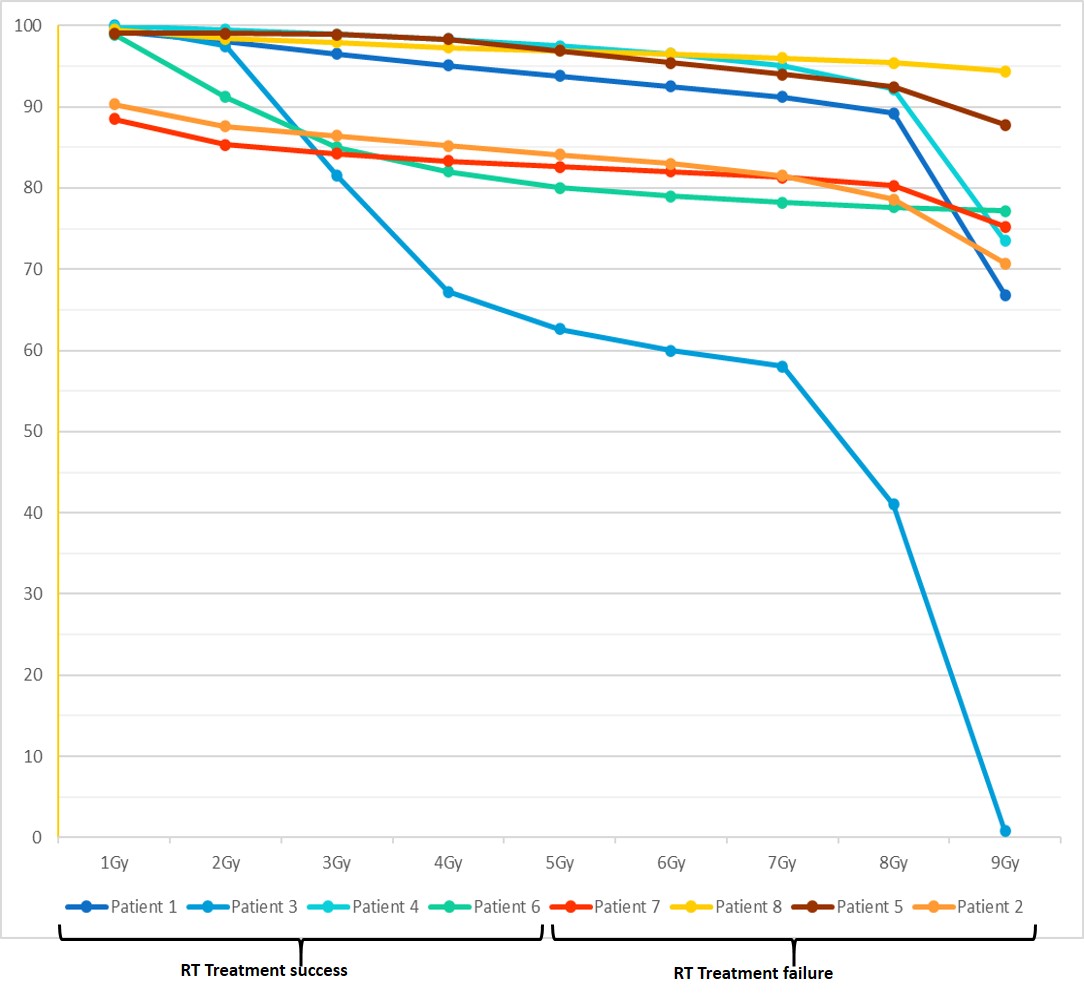


**Figure S1.** Coverage (%) of surgeon-based clinical target volumes (CTVs) at 1–9 Gy isodose levels for eight patients, each represented by a single line. Data are presented descriptively because of the small sample size.


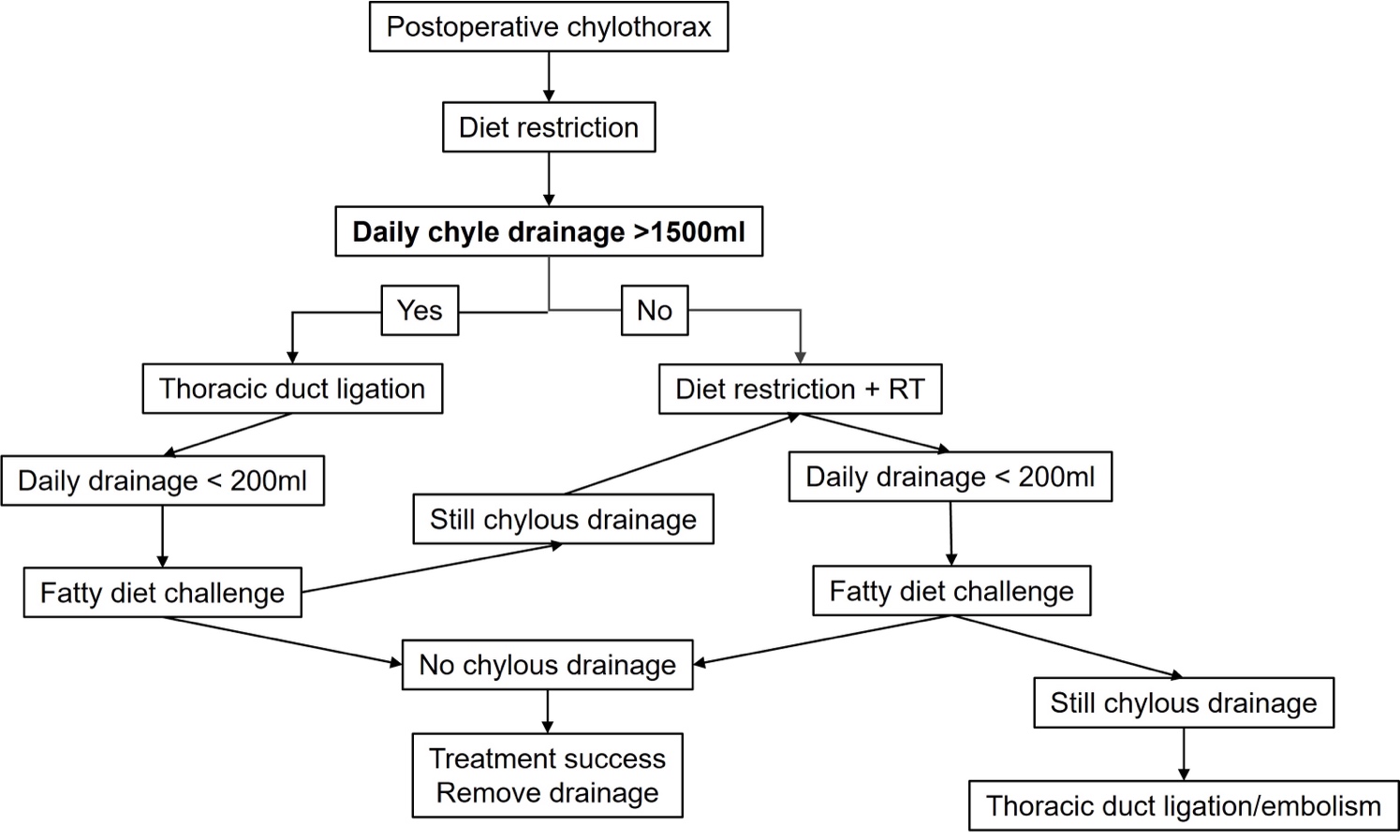


**Figure S2.** Proposed management algorithm for postoperative chylothorax based on daily drainage volume and response to conservative treatment.


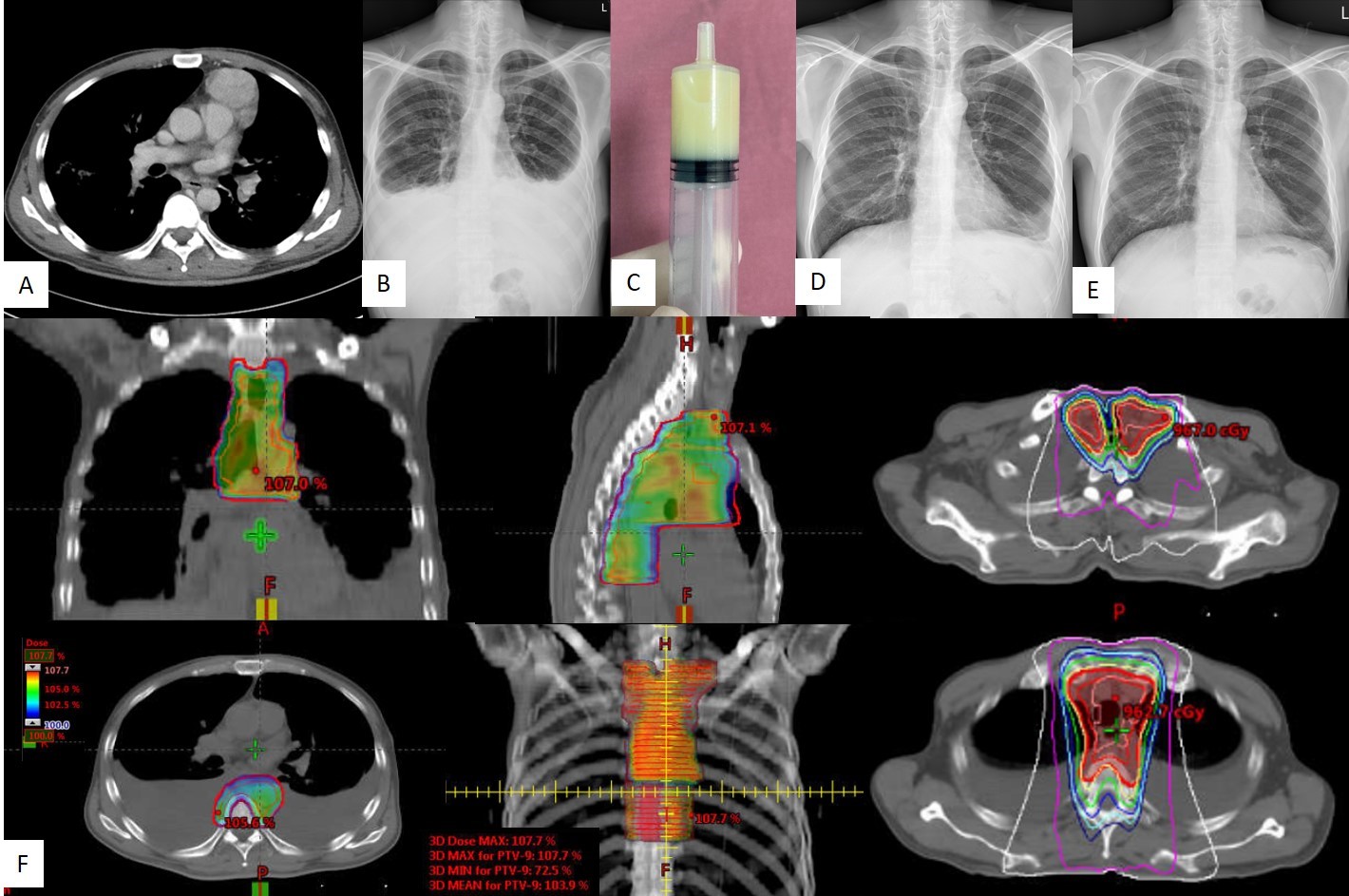


**Figure S3.** (A) Preoperative chest CT demonstrating a large anterior mediastinal mass; (B) Chest radiograph on postoperative day (POD) 8 showing bilateral pleural effusions; (C) Gross appearance of milky pleural effusion consistent with chylothorax; (D) Chest radiograph on post-RT day 16 showing decreased left pleural effusion and resolution of the right effusion; (E) Chest radiograph on post-RT day 30 demonstrating complete resolution of the left pleural effusion; (F) RT planning image illustrating the CTV encompassing the entire dissection area, including the bilateral thymic horns.

# Supplementary Table

| Coverage rate (%)  Patient | 1 Gy | 2 Gy | 3 Gy | 4 Gy | 5 Gy | 6 Gy | 7 Gy | 8 Gy | 9 Gy |
| --- | --- | --- | --- | --- | --- | --- | --- | --- | --- |
| Patient 1 | 99.3 | 98.0 | 96.5 | 96.1 | 93.8 | 93.5 | 91.2 | 89.2 | 66.9 |
| Patient 2 *(failure)* | 99.5 | 98.4 | 97.9 | 97.3 | 96.9 | 96.5 | 96.0 | 95.4 | 88.4 |
| Patient 3 | 100 | 97.5 | 81.5 | 67.2 | 62.6 | 60.0 | 58 | 41 | 0.8 |
| Patient 4 | 99.9 | 99.5 | 98.9 | 98.3 | 97.5 | 96.5 | 95 | 92.2 | 73.5 |
| Patient 5 *(failure)* | 90.3 | 87.6 | 86.4 | 85.2 | 84.1 | 83.1 | 81.5 | 78.6 | 70.7 |
| Patient 6 | 98.9 | 91.2 | 85.0 | 82.0 | 8.01 | 78.0 | 78.2 | 77.6 | 77.2 |
| Patient 7 *(failure)* | 99.0 | 99.0 | 98.9 | 98.3 | 96.9 | 95.4 | 94.0 | 92.4 | 87.8 |
| Patient 8 *(failure)* | 88.5 | 85.3 | 84.2 | 83.3 | 82.6 | 82.0 | 81.3 | 80.3 | 75.2 |

**Table S1.** Radiotherapy (RT) coverage rate at different RT isodose levels (1–9 Gy).
